# Photonic response and temperature evolution of SiO_2_/TiO_2_ multilayers

**DOI:** 10.1007/s10853-021-06557-y

**Published:** 2021-10-07

**Authors:** George Christidis, Olga B. Fabrichnaya, Stefan M. Koepfli, Erik Poloni, Joel Winiger, Yuriy M. Fedoryshyn, Andrey V. Gusarov, Mariia Ilatovskaia, Ivan Saenko, Galina Savinykh, Valery Shklover, Juerg Leuthold

**Affiliations:** 1 Institute of Electromagnetic Fields, ETH Zurich, Gloriastrasse 35, 8092 Zurich, Switzerland; 2 Institute of Material Sciences, Freiberg University of Mining and Technology, 09599 Freiberg, Germany; 3 Department of Materials. ETH Zurich, Complex Materials, 8093 Zurich, Switzerland

## Abstract

The microstructural and optical reflectivity response of photonic SiO_2_/TiO_2_ nanomultilayers have been investigated as a function of temperature and up to the material system’s melting point. The nanomultilayers exhibit high, broadband reflectivities up to 1350 °C with values that exceed 75% for a 1 μm broad wavelength range (600–1600 nm). The optimized nanometer sized, dielectric multilayers undergo phase transformations from anatase TiO_2_ and amorphous SiO_2_ to the thermodynamically stable phases, rutile and cristobalite, respectively, that alter their structural morphology from the initial multilayers to that of a scatterer. Nonetheless, they retain their photonic characteristics, when characterized on top of selected substrate foils. The thermal behavior of the nanometer sized multilayers has been investigated by differential thermal analysis (DTA) and compared to that of commercially available, mm-sized, annealed powders. The same melting reactions were observed, but the temperatures were lower for the nm-sized samples. The samples were characterized using X-ray powder diffraction before DTA and after annealing at temperatures of 1350 and 1700 °C. The microstructural evolution and phase compositions were investigated by scanning electron microscopy and energy-dispersive X-ray spectroscopy measurements. The limited mutual solubility of one material to another, in combination with the preservation of their optical reflectivity response even after annealing, makes them an interesting material system for high-temperature, photonic coatings, such as photovoltaics, aerospace re-entry and gas turbines, where ultra-high temperatures and intense thermal radiation are present.

**Supplementary Information:**

The online version contains supplementary material available at 10.1007/s10853-021-06557-y.

## Introduction

High-temperature photonic materials are attracting more and more attention for applications where thermal stability and optical response as a function of temperature are critical parameters. Among these materials, silicon dioxide (SiO_2_) and titanium dioxide (TiO_2_) have been extensively studied in the visible (VIS) and near-infrared (NIR) parts of the electromagnetic spectrum, since they are widely used in medicine [1, 2], photovoltaics [3, 4], electronics [5, 6] and optics [7–10]. Both oxides display low or no optical losses, they have a high refractive index contrast (*Δn* ~ 1) in the aforementioned electromagnetic spectra and they can be deposited into multilayer coatings using a variety of fabrication methods and techniques.

Both SiO_2_ and TiO_2_ have high melting points (1710 °C and 1843 °C, respectively [11]) and experiments with post-annealed powder mixtures of them have proved that the materials are immiscible in solid state and have limited mutual solubility in liquid state [12, 13]. As a result, they constitute an attractive material couple for high-temperature photonic applications [14], particularly in solar technology [15–17], and have been prepared using different fabrication conditions and methods [18, 19]. At elevated temperature conditions, thermal energy alters the microstructure of each material (phase, density, grain size, etc.), leading to structures different than originally designed. SiO_2_ films transform from amorphous or quartz (low temperature) to cristobalite (~ 900–1400 °C) upon heating depending on the initial crystallinity of the film [20, 21], while in some cases the metastable tridymite is observed especially in the presence of impurities [22, 23]. Conversely, TiO_2_ thin films transform from amorphous to metastable anatase (300–400 °C) and then convert to thermodynamically stable rutile (~ 800 °C) [24, 25]. Recent work has shown that when heated up, a SiO_2_/TiO_2_ multilayer exhibits a slightly different microstructure behavior compared to that of single SiO_2_ or TiO_2_ films. The presence of SiO_2_ increases the phase transition temperature threshold of TiO_2_ and consequently higher temperatures are required to get rutile [26].

In the case of TiO_2_, the phase change does not only lead to improved crystallinity, but also to a refractive index increase from 2.1 (amorphous) up to 2.5–2.7 (rutile) [27, 28], while SiO_2_ retains values close to 1.45 for both amorphous and crystalline films [29, 30]. A refractive index increase also affects *Δn* and thus the photonic response of the whole multilayer stack. This has already been verified for SiO_2_/TiO_2_ multilayers heated up to 500 °C [9]. Upon further heating, a partial melting of the materials and a liquid phase separation is observed. When the temperature reaches values beyond the system’s eutectic point (L = SiO_2_ + TiO_2_, ~ 1550 °C), the liquid, which is in equilibrium with pure solid TiO_2_, can further dissolve TiO_2_ starting from a concentration of 6.3 mol. % (eutectic composition) and further up to SiO_2_ rich liquid in monotectic reaction (26.5 mol. %). In the monotectic reaction point (1780 °C), two liquids with compositions of 26.5 and 86 mol. % TiO_2_ coexist in equilibrium with rutile (monotectic reaction) [11, 31]. At temperatures above the monotectic reaction, two liquids coexist in equilibrium until the critical temperature is reached. The samples, which were held in miscibility gap composition range, typically formed SiO_2_ or TiO_2_ drops inside the other material’s bulk matrix [31, 32]. The microstructure of the final samples is determined by the cooling rate, viscosity, density and interdiffusion of coexisting liquid phases. This high-temperature structural evolution and immiscibility of the SiO_2_/TiO_2_ system makes it an interesting candidate as a photonic additives in thermal protection systems (TPS) and for elevated temperature applications such as thermo-photovoltaics [33], gas turbines [34, 35] and aerospace [36, 37], where high-flux radiation needs to be back-reflected in order to improve a systems’ stability and performance.

Especially in the case of aerospace TPS, reflective photonic additives are required to address the high, incoming thermal radiation flux [38, 39]. This thermal radiation is the result of the space vehicle’s hypersonic velocity during atmospheric entry, which heats up and ionizes the atmosphere’s particles, leading to the formation of a bow shock layer [40]. The ionized particles emit high-intensity radiation at distinct wavelengths, which spread over broad wavelength ranges [41]. It is expected that future exploratory missions to planets like Mars and Venus will experience even higher thermal radiation loads, resulting from the spacecraft’s increased weight and entry velocity [42, 43], and thus enhancing the TPS with photonic inclusions is a possible solution [37].

Recent in and ex situ thermal stability and high-temperature evolution studies have focused only on the mechanical properties of hard multilayer coatings [44–46]. Furthermore, the experiments have been conducted up to temperatures much lower than the melting points of these investigated materials.

In this work, we investigate the high-temperature microstructure evolution and photonic behavior of nanomultilayer SiO_2_/TiO_2_ samples up to 1710 °C. It is shown that they can increase the diffuse reflectivity of absorbing or reflective substrates (e.g., graphite, tungsten) to values exceeding 75% across an almost 1000-nm-wide wavelength range. Further, it is found that the samples display similar microstructure behavior (grain size evolution, phase change temperatures, etc.) as commercially available mm-sized SiO_2_/TiO_2_ powders despite their reduced size features. The successive heating of the nanomultilayers at a temperature of 1350 °C leads to phase transformations, which alter the original microstructure, but not their high reflectivity performance. On the contrary, the reflectivity remains close to the one originally measured. The presented experimental analysis provides a first insight on how microstructural changes affect the nanomultilayers’ photonic response and the potential of the SiO_2_/TiO_2_ material system for high-temperature stable, photonic additives.

## Experimental section

In this section, the fabrication, preparation and characterization (microstructure and optical response) of the SiO_2_/TiO_2_ nanomultilayers and of the reference mm-sized, sintered SiO_2_/TiO_2_ powder samples are briefly presented.

### Design and fabrication of nanomultilayer and reference material

A SiO_2_/TiO_2_ nanomultilayer coating has been designed and optimized by means of an evolution strategy (ES) algorithm. The coating consists of 18 layers with individual layer thicknesses between 60–480 nm for both TiO_2_ and SiO_2_. It has been optimized to provide high and broadband reflectivity in the visible and part of the NIR electromagnetic spectrum (700–1600 nm) as described in [37]. The exact design of the coating can be found in the supplement.

The SiO_2_/TiO_2_ nanomultilayers have been fabricated by Schott AG (Yverdon-Les-Bains, Switzerland) using e-beam deposition. A sacrificial, thin Al_2_O_3_ layer (~ 500 nm) is firstly deposited between the Si substrate and the multilayer coating; Then, the SiO_2_ and TiO_2_ layers are successively deposited with controllable thickness (accuracy 1–2%) at a temperature of 300 °C and a deposition pressure lower than ${10}^{-6}$ mbar. The nanomultilayers are detached from the Si substrate by chemically etching the Al_2_O_3_ layer inside phosphoric acid (H_3_PO_4_) heated at 140 °C for 5 h. Following that, the solution’s pH is neutralized using NaHCO_3_, and then it is filtered to collect the SiO_2_/TiO_2_ nanomultilayers. The amount of coating, collected from a single 4″ Si wafer, equals to approximately 30–32 mg. The acquired nanomultilayers are in a platelet-form with sizes that vary in diameter between 200–300 μm. This is due to the fact that the nanomultilayers are brittle and break into smaller pieces after their removal from the Si substrate.

The reference, mm-sized samples have been prepared by sintering powders of TiO_2_ and SiO_2_ at 1400 °C for a total sintering time of ~ 100 h. The sample has been characterized after the sintering using XRD and SEM/EDX. More specifically, a commercially available SiO_2_ powder (Alfa Aesar, Germany) 99.995% purity having quartz structure, and a TiO_2_ powder from the same producer, purity 99.990% in rutile crystalline form, have been used to create the reference sample. Both powders have a 40 mesh particle size (~ 0.4 mm).

### Microstructural characterization of materials

The thickness, morphology and microstructure of the deposited SiO_2_/TiO_2_ nanomultilayers have been characterized with a Hitachi SU8230 field-emission scanning electron microscope (SEM). For this, the samples have been mechanically cleaved. Energy-dispersive X-ray spectroscopy (EDX) measurements have been performed in order to verify the layers’ elemental composition.

The phase composition and crystallinity of the nanomultilayers have been determined by an X’Pert^3^ Powder (Panalytical). Typical 2*θ* measurement scans have been performed at angles 2*θ* = 15°–85°, where the diffraction peaks of TiO_2_ (anatase, rutile) and SiO_2_ (quartz, cristobalite) are located according to the literature.

The high-temperature behavior of the nanomultilayer samples has been analyzed using differential thermal analysis (DTA). To investigate the phase transformations of the nanomultilayers, two different DTA devices have been used. For temperatures below 1650 °C, a TG–DTA SETSYS Evolution-1750 (SETARAM Instrumentation, France) with a Pt/PtRh 10% thermocouple has been used. The ceramic specimens have been placed in open Pt crucibles, heated up to 1650 °C and cooled in air atmosphere, using a heating and cooling rate of 10 and 30 °C·min^−1^, respectively. DTA measurements at higher temperatures, up to 1850 °C, have been performed in a TG–DTA SETSYS Evolution-2400 (SETARAM Instrumentation, France) using open W crucibles and employing a permanent inert gas flow (He).

Likewise, the microstructure of the mm-sized, reference samples has been examined using a LEO 1530 Gemini, Carl Zeiss (SEM) equipped with an EDX detector (Bruker AXS Mikroanalysis GmbH). Its X-ray powder diffraction (XRD) measurements were performed with a URD63 (Seifert–FPM, Freiberg, Germany) diffractometer using CuK_α_ radiation. In this case, a Rietveld analysis has also been made using MAUD software [47]. The high-temperature behavior of the reference sample was investigated for the sample containing 50 mol.% SiO_2_ and 50 mol.% TiO_2_ under the same temperature and atmosphere conditions. The only difference is that the reference sample has been heated up to 1950 °C in the TG–DTA SETSYS Evolution-2400 system.

## Results and discussion

### XRD characterization of as-deposited nanomultilayers & sintered, reference samples

**Figure 1:**
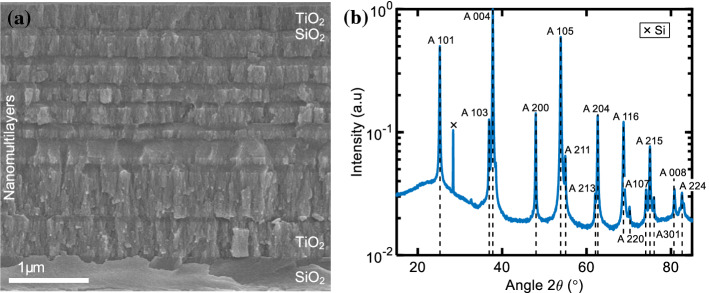
**a** Cross section of SiO_2_/TiO_2_ nanomultilayers, as-deposited on a Si substrate **b** XRD pattern of nanomultilayers after detaching from the substrate. The indexing of anatase TiO_2_ peaks is shown

**Figure 2:**
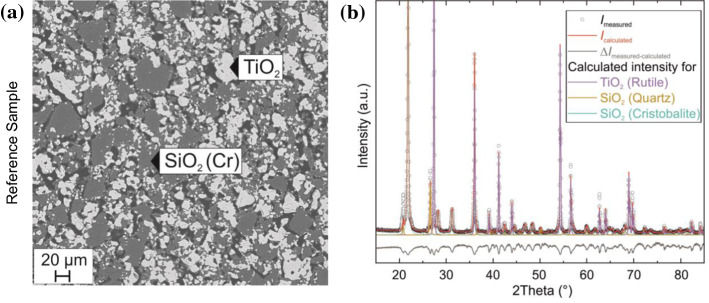
Microstructure **a** and XRD pattern **b** of reference, oxide powder sample sintered at 1400 °C

In this section, we discuss the morphology, phase composition of the as-deposited SiO_2_/TiO_2_ nanomultilayers, after detaching from the substrate, Fig. 1, and compare them to commercially available, sintered, mm-sized SiO_2_/TiO_2_ powder samples, Fig. 2.

The evaporated, nanomultilayer samples consist of SiO_2_ layers that present amorphous phase, and metastable, polycrystalline anatase TiO_2_ layers. The phase composition of the materials is in good agreement with previously published work, since both TiO_2_ and SiO_2_ have been evaporated at slightly elevated substrate temperature (~ 300 °C) [20, 48]. This deposition temperature is well below the crystallization temperature threshold of rutile TiO_2_ (700–800 °C) and quartz SiO_2_ (~ 800 °C). Nevertheless, the intensity of the measured anatase phase differ significantly with respect to the bulk anatase. This can be attributed to the preferred orientation the multilayer displays. This phenomenon has already been reported in the literature [49, 50] and occurs when the crystallites of a sample align parallel to the surface of the measurement holder. The Si peak at 2*θ* = 28.5° can be attributed to small Si chip fragments originating from the substrate during the detaching of the coating from the wafer (cleaving into smaller pieces). In the next step, the Si fragments, which originate from the cleaving of the Si substrate before inserting the sample into the H_3_PO_4_, are removed from the suspension using suction filtration. The cleaving of Si into smaller pieces is necessary to speed up the wet etch process. The fragments themselves do not contribute to the photonic performance of the sample, but could contaminate it.

Figure 2a, b shows the microstructure and XRD pattern of the reference SiO_2_ and TiO_2_ powder, sintered at 1400 °C, for comparison. It can be concluded based on EDX measurements that there is no mutual solubility between TiO_2_ and SiO_2_, i.e., rutile TiO_2_ does not dissolve any SiO_2_ and vice versa. The sintered sample displays distinct SiO_2_ and TiO_2_ regions with maximum dimensions up to approximately 40 μm. The microstructure of the annealed sample is in accordance with previous work [31] and stems from the immiscibility of the two materials. The sintered SiO_2_/TiO_2_ sample does not exhibit distinct structural morphology features and the materials are already in their high-temperature, stable phases. It should be noted that this annealed, heterogeneous sample has grains with relative interfaces much larger than the nanomultilayer samples presented in Fig. 1a. An EDX analysis reveals that the reference, annealed oxide powder samples form μm–sized grains, when heated at 1400 °C, which consist of SiO_2_ (8 vol. % quartz and 56 vol. % cristobalite) and 36 vol. % TiO_2_ rutile. The results for both nanomultilayers and reference sample are summarized in Table 1.

**Table 1:** Weight ratio and vol. % of nanomultilayers and mm-sized, annealed powder sample

	SiO_2_ layer – Weight ratio %	TiO_2_ layer – Weight ratio %
Nanomultilayers	Si: 73.08	Ti: 40.34
	O: 26.92	O: 55.64
		Si: 4.02
Reference, sintered powders	SiO_2_ – Vol. %	TiO_2_ – Vol. %
	Quartz: 8	Rutile: 36
	Cristobalite: 56	

### DTA and microstructure evolution as a function of temperature

**Figure 3:**
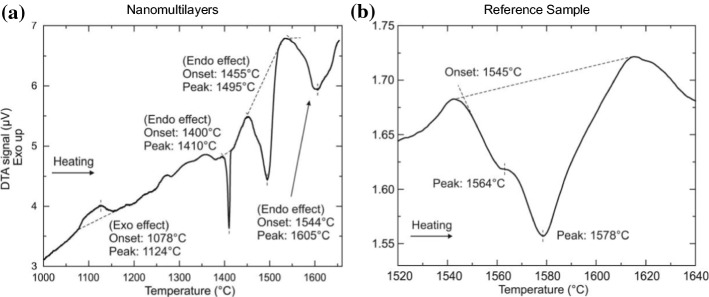
Differential thermal analysis (DTA) curve of **a** nanomultilayer sample and of **b** reference, sintered sample heated up to 1650 °C

A DTA for both samples has been performed in air atmosphere up to 1650 °C and in inert He atmosphere up to 1850 °C for the nanomultilayer sample and up to 1950 °C for the reference powder. For investigations up to 1650 °C, a Pt crucible in air atmosphere has been chosen. In order to investigate transformations above the melting point of Pt, tungsten crucibles have been used. Consequently, to avoid the oxidation of these W crucibles the experiments have been performed in inert gas atmosphere (He). The results of the DTA investigation for both sample in air are presented in Fig. 3a, b.

The DTA of the nanomultilayers, Fig. 3a, first reveals a wide exothermic effect in the temperature range between 1078 and 1150 °C, followed by an endothermic heat effect observed at a temperature of 1400 °C, a second larger endothermic effect at 1455 °C and a smaller third effect at 1544 °C. Conversely, the reference, sintered oxide sample only shows a broad, complex endothermic effect caused by at least two transformations. The temperature of the first onset was around 1545 °C, Fig. 3b. Using the phase diagram of the TiO_2_-SiO_2_ system [31, 32], the first effect can be explained by the eutectic reaction (Liquid = SiO_2_ + TiO_2_), followed by a continuous melting in two-phase field (Liquid + TiO_2_)_._

**Figure 4:**
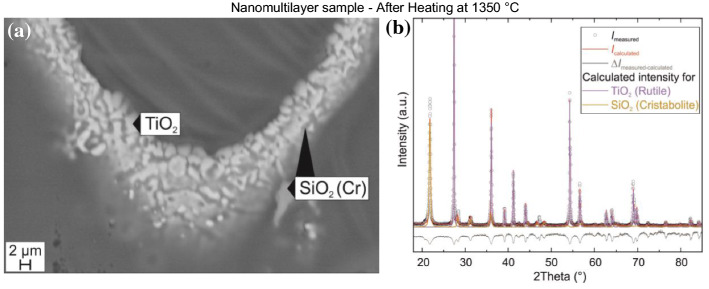
**a** The microstructure of the multilayered sample after heat treatment at 1350 ºC revealing TiO_2_ grains (white) in an SiO_2_ matrix (gray). The dark area, around the sample is resin. **b** XRD of the nanomultilayer sample after heat treatment at 1350 °C

The exothermic effect of the nanomultilayers can be explained by the anatase to rutile transformation of TiO_2_ and the anticipated crystallization of amorphous SiO_2_ to quartz and cristobalite. In order to  verify the origin of this exothermic effect the nanomultilayers are heat-treated at 1350 °C. The microstructure of scales obtained after heat treatment was investigated using SEM, Fig. 4a. The XRD pattern after heating is presented in Fig. 4b. The microstructure of the nanomultilayers shows that the nm layers have completely disappeared and TiO_2_ grains of approximately 2 μm in size have formed inside a SiO_2_-rich matrix. Additionally, separate SiO_2_ grains can been observed. The XRD pattern of the samples indicates the presence of rutile (TiO2) and cristobalite (SiO2), which are well fitted by Rietveld analysis. Additional diffraction peaks have been observed at 2*θ* = 28.5° and 2*θ* = 48°. These peaks have a higher intensity than the ones expected for cristobalite and do not correspond to the peak positions of α-quartz (2*θ* = 26.6° and 2*θ* = 50°). Therefore, since no other elements except Ti, Si and O have been detected by EDX, these peaks can only be explained by the presence of metallic Si (2*θ* = 28.1° and 2*θ* = 47.4°). The presence of cristobalite indicates that silicon dioxide melting did not occur, because after melting a SiO_2_-rich, glassy-type amorphous phase would have formed. Glassy materials would appear as broad background peaks at low 2*θ* values in an XRD pattern. Hence, the exothermic effect is due to the phase transformation to the thermodynamically stable cristobalite (SiO_2_) and rutile (TiO_2_) phases. The temperature range in which these phase changes occurred is consistent with [26].

In the nanomultilayer sample one can further observe three endothermic peaks. The first and second heat effects are distinct and are observed at 1400 and 1455 °C, respectively, Fig. 3a. The third effect is less sharp and occurred at 1544 ºC. We assume that the first endothermic peak (1400 °C) is due to the melting of Si. To confirm this assumption, the sample has been heat treated at 1420 °C and successively characterized by SEM and XRD. It is found that after the 1420 °C heat treatment, the XRD pattern is similar to the one obtained after the heat treatment at 1350 °C, presented in Fig. 4b, characterized by a higher intensity of peaks at 2*θ* = 28.5° and 2*θ* = 48°. It is possible that the Si distribution was not homogeneous in the sample and the portion heat treated at 1420 ºC contained more Si. Hence, the Si chip traces, which had been initially detected by the XRD, have not oxidized during heating in DTA, even when heated at 1420 °C. However, after the heat treatment at 1650 °C Si does not remain in metallic state. It melts and becomes part of the SiO_2_/TiO_2_ sample. Moreover, the microstructure of the nanomultilayers, heat-treated at 1420 °C, is similar to the one observed at 1350 °C, Fig. 4a. Consequently, the melting of Si has no effect on the nanomultilayer sample. The second heat effect at 1455 °C is related to the eutectic reaction Liquid = SiO_2_ + TiO_2_. It should be noted that the onset temperature of this second effect is 90 °C lower in the nanomultilayer case than that of the reference, sintered sample, which has an onset of 1545 °C. This can be explained by the significantly smaller TiO_2_ grains size in the multilayer sample compared to the sintered, reference powders. The third effect at 1544 ºC in the multilayer sample can be explained by the continuous melting in two-phase Liquid + TiO_2_.

**Figure 5:**
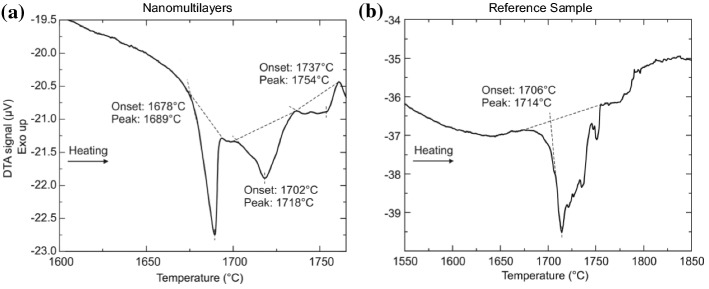
**a** Differential thermal analysis (DTA) of nanomultilayers heated up to 1780 °C under a He atmosphere. **b** DTA curve of reference, sintered samples heated up to 1850 °C under the same conditions

Figure 5a, b presents the DTA of the samples at a temperature of up to 1850 °C, under a He atmosphere. It shows a large endothermic effect at 1678 °C for the nanomultilayer and at 1706 °C for the sintered, reference sample.

The endothermic effects at 1678 °C (nanomultilayers) and 1706 °C (reference) can be explained by the liquid separation according to the monotectic reaction L_2_ = L_1_ + TiO_2_. It should be noted that for both samples these heat effects have a quite complex structure and the difference in onset temperatures is less than 30 °C (~ 28 °C).

**Figure 6:**
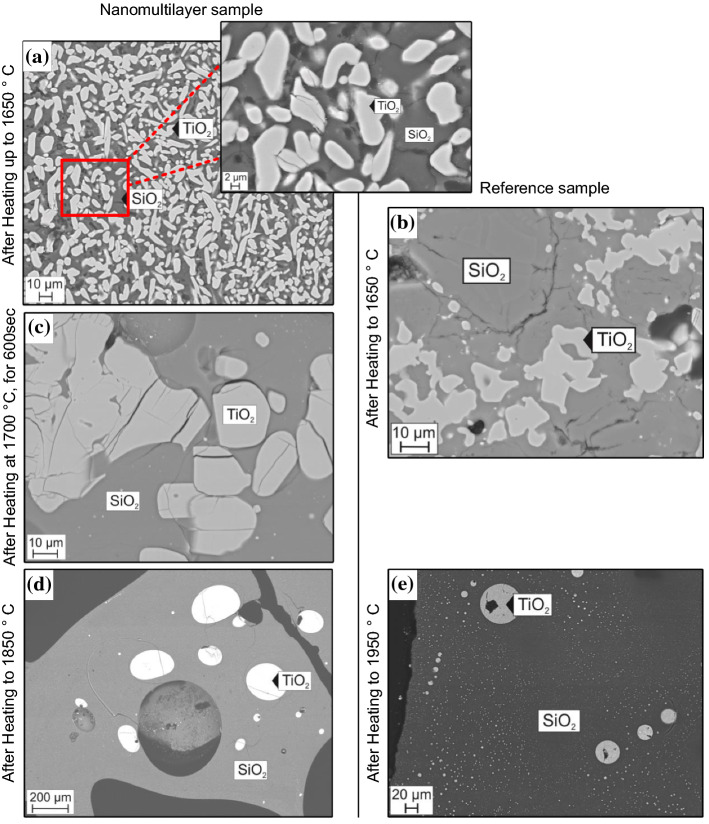
**a** Microstructure of the nanomultilayer sample after heating up to 1650 °C in DTA and its inset. **b** Microstructure of reference, sintered sample heated up to 1650 °C **c** Nanomultilayers’ microstructure after heat treatment at 1700 °C in DTA for 600 s. **d-e** Microstructure of the nanomultilayer sample after heating up to 1850 °C and of the reference sample after heating up to 1950 °C, respectively

In order to better understand the thermal behavior of both samples, their microstructure after the heating in DTA has been investigated. Additionally, SEM images of both samples have been collected after heating up to the temperatures below or greater than the onsets of heat effects observed in DTA, Fig. 6. More specifically, Fig. 6a presents the microstructure of the nanomultilayer sample after heating up to 1650 °C in DTA, while Fig. 6b shows the microstructure of the reference sample after heating in DTA under the same conditions. In both cases, TiO_2_ grains inside a SiO_2_-rich matrix can be observed but with different sizes. In the nanomultilayers, the grains are substantially smaller (~ 2 μm) and homogeneously distributed in the SiO_2_ matrix, Fig. 6a and inset, while in the reference sample the TiO_2_ grains have sizes exceeding 10 μm, Fig. 6b. The much smaller grain sizes of the nanomultilayer sample can explain the difference in thermal behavior with the reference sample.

An SEM/EDX analysis of the samples’ images after heating at 1650 °C, see Fig. 6a, shows that the white TiO_2_ grains do not contain SiO_2_ (~ 1 mol. %), while the dark SiO_2_ areas contain TiO_2_ corresponding to a eutectic composition (7 mol. % TiO_2_). This indicates that the TiO_2_ grains, which were partially melted by the eutectic reaction TiO_2_ + SiO_2_ → L_1_, enriched the liquid with TiO_2_, while unmelted TiO_2_ grains preserve their pure composition.

To understand the reason of the first effect observed during heating in DTA up to 1850 ºC, the nanomultilayer sample was heated up to 1700 ºC in DTA and held in that temperature for 600 s, Fig. 6c. A substantial change of microstructure can be observed. Large grains of TiO_2_, in the range approximately 20–70 μm, in a SiO_2_-rich matrix and small droplets of molten TiO_2_ are present after this heat treatment. The grain size of TiO_2_ is even higher than that of the reference sample heated up to 1650 ºC, Fig. 6b. Upon further heating, the nanomultilayer sample exhibits a second heat effect at the same temperature as that of the reference sample (~ 1700 °C). This monotectic reaction first affects the small TiO_2_ grains, while at the same time unmelted TiO_2_ starts to form larger grains. Figure 6d shows that the monotectic reaction was completed upon heating the nanomultilayer sample up to 1850 °C. Microstructure characterization after heating up to 1850 °C for the nanomultilayers, Fig. 6d, and up to 1950 °C for the reference sample, Fig. 6e, indicates a similar microstructure that is typical of liquid separation for both samples. According to the SEM/EDX analysis a liquid separation occurred with the white drops of frozen TiO_2_-rich liquid containing 10% mol. SiO_2_ and the dark SiO_2_-rich liquid containing 16 mol. % TiO_2_. It should be noted that the compositions of TiO_2_-rich liquid and SiO_2_-liquid were the same within uncertainty limits measured in the multilayer and in the reference sample.

### Optical response evolution of nanomultilayers as a function of temperature

In this section, we evaluate the photonic enhancement response of the SiO_2_/TiO_2_ nanomultilayers as a function of temperature and microstructural change. Additional information about the sample’s characteristics and optical microscopy images of them can be found in the supplement.

**Figure 7:**
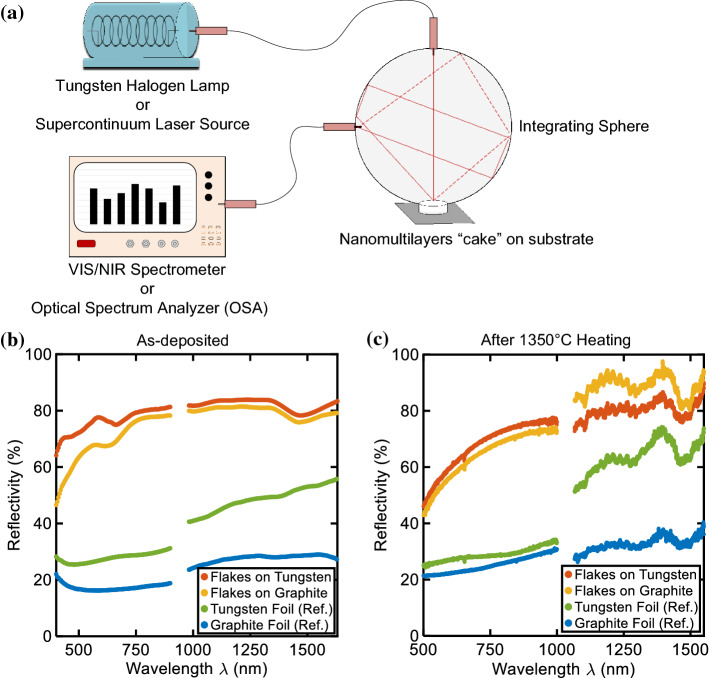
**a** Scheme of optical characterization setup utilizing an integrating sphere. **b** Directional-hemispherical reflectivity measurements of the SiO_2_/TiO_2_ nanomultilayers placed onto a tungsten base (red) and graphite base (orange) at room temperature and **c** after heating up to a temperature of 1350 °C as a function of wavelength. The reference reflectivities of the tungsten and graphene bases (without platelets) are shown by the solid green and blue lines

For the optical characterizations, the nanomultilayer flakes are mixed with ethanol and are being placed into cylindrical containers. The base of the container comprises a substrate (e.g., graphite, tungsten). After the ethanol has evaporated, the container is being removed. The sample (“cake”) has a height of about ~ 2–2.2 mm and a radius of 6 mm. Then the “cake” is controllably brought to the bottom part of an integrating sphere (ISP-50–8-R – Ocean Insight), where its directional-hemispherical reflectivity is measured. More particularly, light generated from a tungsten-halogen lamp (HL-2000, Ocean Insight) or a supercontinuum laser source (SuperK Red, KOHERAS/NKT Photonics) is coupled to an optical fiber and illuminates the top of the sample. The scattered, reflected light is collected by a second optical fiber and is measured either at an optical spectrometer (Flame VIS–NIR Spectrometer, Ocean Insight / CCS200M, ThorLabs) or at an optical spectrum analyzer (OSA – Yokogawa AQ6370C). A diffuse reflectivity standard (WS1–PTFE, Ocean Insight) has been used as a reference in order to evaluate the directional-hemispherical reflectivity. A scheme of the used optical setup is shown in Fig. 7a. The photonic response of the powder is measured with different substrate bases having known optical properties. More specifically, we use tungsten, which is partially reflecting and absorbing and graphite, which is highly absorbing across the whole wavelength range of interest. Fig. 7b presents the as-deposited reflectivity of the nanomultilayers on top of the two substrates, while Fig. 7c shows the reflectivity after heating up at 1350 °C. The pure substrate reflectivity is also provided as a reference.

The directional-hemispherical reflectivity of the nanomultilayer flakes and the substrate bases are plotted in Fig. 7b, c. Fig. 7(b) shows the photonic response at room temperature. It can be seen that the reflectivities of reference substrate, comprising a tungsten base or graphite base, increase from a value of ~ 26–56% (solid green line) and ~ 16–29% (solid blue line) to values close to 80%, when the flakes are added. The nanomultilayers exhibit a high reflectivity response in the range between 850 and ~ 1400 nm, which closely matches the window of interest for protection against thermal radiation during atmospheric re-entry (700–1600 nm). Their response does not display pronounced reflectivity dips (resonances), in the aforementioned range, and is relatively flat. The reflectivities of the materials measured at room temperature, see Fig. 7b, and the reflectivities of the materials after annealed at 1350 °C SiO_2_/TiO_2_, see Fig. 7c, show comparable values independent of the base substrate. The transformation of both SiO_2_ and TiO_2_ into their thermodynamically stable phases at higher temperature leads to a material density change, which manifests as a change in the reflectivity response shown when going from Fig. 7b,c. Nevertheless, the overall performance of the samples does not dramatically change between the as-deposited and after heating up measurements. It should be noted that the characterization using an integrating sphere accounts for all different angles of incidence (directional–hemispherical reflectivity) and as such any reflectivity dips or peaks are smoothened. The similarity of the reflectivity curves for both temperatures and substrate types, in combination with the intrinsic, optical properties even with different substrate bases, demonstrates that reflectivity is mainly the result of the multilayer powder and not the substrate bases. Therefore, it can be concluded that the optical thickness of the measured “cakes” is in each case much greater than unity. The slight absolute difference between the two reflectivity curves (nanomultilayers on graphite and tungsten) may be attributed to some randomness in the surface morphologies of the “cakes”, measurement inaccuracies and variations in the positioning of the “cake” at the opening of the integrating sphere, supplement. These morphological variances are due to the fact that the nanomultilayers are originally in a fine powder form (as-deposited) and in a grainy powder form (after heating at 1350 °C). Consequently, they exhibit high electrostatic forces, are brittle and require the use of ethanol for handling (“cake” preparation and transfer from one substrate to another). As a result, even though the “cake” samples appear similar in shape, thickness and morphology, they are not identical to one another during consecutive measurements on different substrates and at different temperatures. Another important point is that the two samples, as-deposited and after heating at 1350 °C, have been measured in different characterization setups, using a variety of sources and detectors. To that extent, some experimental error is expected as for example with the tungsten foil reflectivity, which shows a “jump” around 1 μm (green curve). Even though similar behavior has been observed in literature for tungsten [51, 52], in our case the change is more pronounced and it can be attributed to additional measuring error found in both as-deposited and heated sample. Additionally, there is a profound ripple effect present in the NIR part of the spectrum of the heated at 1350 °C sample due to the supercontinuum laser source.

## Conclusions

The high-temperature characteristics of SiO_2_/TiO_2_ nanomultilayers demonstrated that the materials are interesting as a photonic additives for next generation photonically enhanced TPS. In view of this application, the microstructural and photonic response evolution of SiO_2_/TiO_2_ nanomultilayers, have been investigated as a function of heating temperature. It has been shown that a powder of SiO_2_/TiO_2_ nanomultilayers has increased the diffuse reflectivity of the underlying substrate base to values exceeding 75% across the whole VIS and part of the NIR electromagnetic spectrum. This has been proven independent of the used substrate, i.e., the same photonic performance is observed for a highly absorbing (graphite) or a weak reflecting (tungsten) substrate base. The nanomultilayers retain their optical characteristics upon heating up to 1350 °C, despite the microstructure changes they undergo when heated. This microstructural evolution with temperature is barely dependent on their reduced grain size and exhibits lower phase transitions temperatures to that of the reference, sintered at 1400 °C cmmercially available mm-sized powder. This has been verified by DTA, SEM/EDX and XRD characterization. The SiO_2_ and TiO_2_ immiscibility to one another in solid state and liquid separation with limited mutual solubilities, in combination with their thermal, microstructural behavior, constitute a prospective material system for the large scale fabrication of photonic additives that can be prepared by a variety of methods and techniques in quantities and dimensions compatible with TPS technology. These SiO_2_/TiO_2_ nanomultilayers are engineered to possess desired photonic responses in desired wavelength bands at both low and high temperatures, making them a better option compared to commercial powders.

## Supplementary Information

Below is the link to the electronic supplementary material.

Supplementary file1 (DOCX 51996 kb)
