# Supplementary material for: Photonic response and temperature evolution of SiO2/TiO2 multilayers
Source: J Mater Sci. 2021 Oct 7;56(33):18440–52. doi: 10.1007/s10853-021-06557-y (PMC8550000; doi:10.1007/s10853-021-06557-y)
Supplement: Supplementary file 1 — Supplementary file1 (DOCX 51996 kb) [file 10853_2021_6557_MOESM1_ESM.docx]

**Supplement**

1. **Nanomultilayer Coating – Design**

In this section, the nanomultilayers’ thicknesses are briefly presented. Figure S1 shows a graphical scheme of the coating design. It consists in total of 18 SiO_2_/TiO_2_ layers, whose thickness have been selected by means of an Evolution Strategy (ES) algorithm, implemented in Matlab (MathWorks, Inc.). The coating has been optimized to be broadband reflective in the NIR part of the electromagnetic spectrum on top of metallic substrate. The individual layer thicknesses vary between 50 and 500 nm.

Fig. S1. Scheme representation of nanomultilayer coating.

Table S1. Thickness of nanomultilayer coating in nm.

| *Layer* | *Material* | *Thickness, nm* |
| --- | --- | --- |
| h_1_ | SiO_2_ | 380 |
| h_2_ | TiO_2_ | 385 |
| h_3_ | SiO_2_ | 65 |
| h_4_ | TiO_2_ | 480 |
| h_5_ | SiO_2_ | 100 |
| h_6_ | TiO_2_ | 60 |
| h_7_ | SiO_2_ | 230 |
| h­_8_ | TiO_2_ | 75 |
| h_9_ | SiO_2_ | 90 |
| h_10_ | TiO_2_ | 160 |
| h_11_ | SiO_2_ | 80 |
| h_12_ | TiO_2_ | 125 |
| h_13_ | SiO_2_ | 85 |
| h_14_ | TiO_2_ | 175 |
| h­_15_ | SiO_2_ | 120 |
| h_16_ | TiO_2_ | 215 |
| h_17_ | SiO_2_ | 100 |
| h_18_ | TiO_2_ | 250 |

1. **Optical Measurements – Inaccuracy**

In section 3.3, optical measurements of the as–deposited nanomultilayers and from the sample after heating up to 1350 °C have been presented. As it can be observed from Fig. 7 (b) and (c), even though the graphs are similar in form there seems to be a small reflectivity “jump” between the VIS (550–980 nm) and NIR (1000–1600 nm). The rapid reflectivity change, in such a small wavelength range, cannot be explained by the causality principle and hence it can be considered an effect of measurement inaccuracy. More specifically, as it has been already mentioned, the nanomultilayers’ sample is prepared into a powder “cake” and brought to the bottom part of the integration sphere for the optical characterization. Even though the sample preparation procedure is the same for both substrates (tungsten and graphite) and measurements (VIS and NIR), small deviations of the sample’s morphology and positioning are possible. As a result, the sample’s reflectivity can be overestimated with respect to the intensity of the reference signal. Fig. S2 presents the ideal measurement case (a) and some possible measurement overestimation scenarios (b), (c).

Fig. S2. Optical measurement inaccuracy scenarios during an integration sphere measurement. (a) Ideal scenario. (b) Measured sample partially entering the outer part of the sphere. (c) Measured sample partially entering the outer part of the sphere and/or being non flat/uniform.

In a reflectivity integrating sphere measurement one aims to bring the measured sample and reference close to the inner part of the sphere in order to complete it and acquire the maximum signal intensity. Yet, the physical opening of the sphere limits the size of the sample/reference that can be used and for that reason it is common to use the outer point of it as a positioning point, Fig. S2(a) [1] . By using this approach, one underestimates the intensity of the collected signal as it has already been reported in [2]. In this work, and in order to have the same position reference, all optical measurements have been conducted with the sample and reference being in contact to the outer part of the integration sphere, Fig. S2(a). Nevertheless, the loosely as–deposited SiO_2_/TiO_2_ nanomultilayers and the grainy sample after heating up to 1350 °C may have be placed between the inner and outer part of the integration sphere because of positioning, Fig. S2(b), or the sample may not be completely flat after drying, Fig. S2(c). In that case, the collected signal from the sample is greater than expected and its reflectivity overestimated with respect to the intensity of the reference’s collected signal. Therefore, the reflectivity “jumps” are not so significant into evaluating the reflectivity of the additives as a function of temperature and the measurements have a greater qualitative than quantitative meaning.

1. **Laser Confocal & Optical Microscopy**

To evaluate the microstructure of the additives’ sample (“cake”) after heating at 1350 °C, optical microscopy images are collected using a Laser Confocal Microscope, Fig. S3, and a HD digital microscope camera (Leica MC170HD), Fig. S4.

Fig. S3. Laser confocal microscopy images of nanomultilayers after heating at 1350 °C. (a)-(f) Images collected with a 10x magnification at two different locations of the sample with varying focus planes, Location 1 (a-c) and Location 2 (d-f). Top view image of the sample at a 2x (g) and 20x (h-i) magnification.

From Fig. S3 (a-c) and (d-f) one can easily observe that there are distinct planes of additives parallel to the top surface of the sample. More specifically, Fig. S3(b) is collected at a plane 20 μm lower than Fig. S3(a), while Fig. S3(c) 40 μm. Fig. S3(e) and (f) are similarly collected from planes lower by 20 and 40 μm to that of Fig. S3(d). In both cases, it can be observed that the nanomultilayer flakes have irregular shapes in the order of 200-250 μm in diameter, but also smaller pieces are present. When one increases the magnification, Fig. S3(h-i), then it is possible to observe smaller regions of one material (spots) inside the other material. This is consistent with the SEM images analysis presented in the main text, Fig 6. Top view images of the sample have also been collected, Fig. S4.

Fig. S4. Top view images of nanomultilayers’ sample after heating at 1350 °C under different magnifications, 1.6x-(a), 3.2x-(b), 5x-(c).

The images indicate that most of the nanomultilayers’ powder is white with some yellow particles due to sub-stoichiometric SiO_2_ ­and/or TiO_2 ­­­_material during the 1350 °C heating (under inert atmosphere). Small contamination (hair fiber and graphite pieces) are present because of handling and transferring between the two substrates. The “cake” seems to be reflective in the VIS part of the camera (white color), the additives are aligned at different levels and are, up to a certain degree, parallel to the top surface of the sample. The relative size of individual platelets appears to be up to 300 μm and their shape is irregular due to the preparation process.

**References**

1. Clarke FJJ, Compton JA (1986) Correction methods for integrating-sphere measurement of hemispherical reflectance. Color Research & Application 11 (4):253-262. doi:<https://doi.org/10.1002/col.5080110406>

2. Zerlaut GA, Anderson TE (1981) Multiple-integrating sphere spectrophotometer for measuring absolute spectral reflectance and transmittance. Appl Opt 20 (21):3797-3804. doi:<https://www.doi.org/10.1364/AO.20.003797>
